# Adaptation and Validation of the Multi-Dimensional Perceived Autonomy Support Scale for Physical Education to the Spanish Physical Exercise Context

**DOI:** 10.3390/ijerph17113841

**Published:** 2020-05-28

**Authors:** Ruben Trigueros, José M. Aguilar-Parra, Ana I. Sánchez-Iglesias, Jerónimo J. González-Bernal, Isabel Mercader

**Affiliations:** 1 Department of Language and Education, University of Antonio de Nebrija, 28015 Madrid, Spain; 2 Department of Psychology, Hum-878 Research Team, Health Research Centre, University of Almería, 04120 Almería, Spain; 3 Department of Health Sciences, Cavidito Research Team, Health Research Centre, University of Burgos, 09001 Burgos, Spain; asiglesias@ubu.es (A.I.S.-I.); jejavier@ubu.es (J.J.G.-B.); 4 Department of Psychology, University of Almería, 04120 Almería, Spain

**Keywords:** adolescence, autonomy support, validation, factor analysis, physical exercise

## Abstract

The interaction between the teacher and the student is essential in order to encourage adherence to physical exercise or sports by young people. In this sense, the support of the autonomy of the teacher for the students has been analyzed in a one-dimensional way. Therefore, the aim of this study was to adapt and validate the Multidimensional Scale of Support for Autonomy Perceived for Physical Education the context of Spanish to physical exercise, in order to have a multidimensional scale. A total of 2329 young people from various educational centers in Andalusia (Spain) participated in the study. The factorial structure of the questionnaire was examined through an exploratory factorial analysis and two confirmatory factorial analyses. In addition, an analysis of invariability by sex and age was carried out. The results reflected that the validated questionnaire showed adequate psychometric properties, being invariable with respect to sex and age.

## 1. Introduction

The influence of the teacher on Physical Education (PE) students is fundamental, since he or she contributes to the adoption of a series of habits related to the practice of fiscal and/or sports exercise by young people during their free time [1]. Therefore, the leisure and free time habits of young people depends, to a greater or lesser extent, on the teacher’s ability to relate effectively to his or her students, to motivate them and to create a positive classroom climate towards the practice of physical activity [2]. In this sense, different studies have shown that support for teacher autonomy is related to positive emotional experiences, the development of well-being and the academic performance of students [3,4]. However, most of these studies that have analyzed teacher autonomy support in relation to physical exercise have been based on a one-dimensional scale called the Perceived Autonomy Support Scale for Exercise Environments (PASSES) [5]. However, at present Stefanou et al., [6] have proposed that autonomy-supporting behavior could be characterized by three dimensions (organizational, cognitive and procedural), providing a better understanding of the teacher’s behavior and the students’ adaptation processes towards PE classes. Thus, this study aims to adapt and validate the Multidimensional Scale of Perceived Autonomy Support for Physical Education of Tilga et al. [7] to the context of Spanish physical exercise.

Self-determination theory [8] suggests the influence of social context as an essential element in the development of individuals towards their own personal, psychological and emotional growth [9,10]. In this way, support for autonomy is understood as the disposition contrary to the position of authority, such as taking the perspective of others, providing appropriate and meaningful information, facilitating decision-making and minimizing the use of coercive means [9]. Studies to date have been based on a one-dimensional scale of autonomy support called PASSES by Hagger et al. [5], showing a positive relationship with basic psychological needs [11,12], positive emotions [13,14], autonomous motivation [15,16], learning experience [17,18] and coping strategies [19,20].

This scale, however, only captures the cognitive aspect of teacher’s autonomy support, ignoring the other factors that would allow a more complete and holistic understanding of the teacher’s influence which underlies the optimal behavior and learning of the students [6,11]. These factors would be the (a) organizational, (b) procedural and (c) cognitive dimensions. The first dimension refers to the teacher’s encouragement of the students’ ownership of decisions in relation to physical activity (e.g., location of exercises, choice of the type of physical exercise or sport, etc.). The second dimension refers to the teacher’s encouragement of the learning and assimilation of skills through physical exercise or sport of the students (e.g., the teacher’s ability to learn from students’ physical experiences and the opportunity to select the best way to demonstrate competence). The third dimension refers to the teacher’s promotion of the students’ own ownership of their mental processes in making decisions and solving problems on the basis of their own inventiveness (e.g., freely discussing ideas, re-evaluating decisions, asking questions and having time to make decisions) [6,21].

Based on the theoretical assumptions of Stefanou et al. [6], Tilga et al. [7] developed, created and validated the Multi-Dimensional Perceived Autonomy Support Scale for Physical Education in order to provide researchers with a direct tool to assess autonomy support in a multifactorial way. To this end, they carried out three studies in which they analyzed the factor structure of the questionnaire [7]. In the first of the studies, 62 high school students took part and were administered a 49-item questionnaire in order to determine whether the items were correctly understood. Subsequently, the set of items was analyzed by four researchers from the PE class field, who determined that 12 of the items presented some problems (e.g., overlap and indetermination). In the second of the studies, 1152 high school students participated, where the factor structure of the questionnaire was analyzed through exploratory factorial analysis (EFA) and confirmatory factorial analysis (CFA). The EFA showed that the factor structure of the questionnaire was made up of 21 items distributed among three factors, with 16 items being eliminated since the item–test correlation was below 0.30. Regarding the confirmatory factor analysis, the factor structure of the questionnaire reflected that it was composed of 15 items distributed among three factors, with six items being eliminated since the standardized multiple residues exceeded ± 2.00. In addition, the questionnaire, through an invariance analysis, was shown to be invariant with respect to age and sex. In the third study, 262 high school students participated, where the predictive validity of the scale was analyzed. The results showed that each of the three factors supporting autonomy was positively associated with the satisfaction of psychological needs. This scale has also been validated towards the Spanish context of PE by Burgueño et al. [22]. The authors performed both exploratory and confirmatory factorial analyses with independent samples, showing adequate psychometric properties and a three-factor structure for the questionnaire. In addition, they performed a gender invariance analysis, showing that each of the items is understood in a similar way by boys and girls.

Based on this background, the aim of the present study was to analyze the psychometric properties of the Multi-Dimensional Perceived Autonomy Support Scale for Physical Education of Tilga et al. [7], in order to adapt and validate it to the Spanish physical exercise context. The hypotheses put forward are as follows: (a) both the EFA and the CFA show that the structure of the questionnaire is composed of three factors that correlate positively; (b) the questionnaire shows evidence of reliability; (c) the structure is invariant with respect to gender and age.

## 2. Methods

### 2.1. Participants

The participants in the study comprised 2329 students belonging to various secondary schools from Andalusia (Spain). The sampling that was followed was non-probabilistic incidental, based on the secondary schools to which we had access.

A sample of 847 students (449 males and 398 females) aged 14–18 years was used for the EFA (M = 15.76; SD = 1.06). For the CFA, we used the remaining sample of 1482 students (834 males and 648 females) aged 13–18 (M = 15.36; SD = 1.15).

All students participated in Physical Education classes. Of the students who participated in the study, 57.14% did some kind of sport or physical exercise outside of PE classes.

### 2.2. Measurements

For the scale of support for autonomy in the sports context, the Multi-Dimensional Perceived Autonomy Support Scale for Physical Education by Tilga et al. [7] was used. This scale is composed of 15 items equally distributed among three factors: organizational dimension, procedural dimension and cognitive dimension, as shown in Appendix A. Each of the items on the scale is answered using a Likert-type scale that ranges from 1 = strongly disagree to 7 = strongly agree.

### 2.3. Procedure

The questionnaire was first translated from English into Spanish following the strategy of Bartram et al. [23]. This procedure consisted of translating the scale from English to Spanish by a group of translators with more than 12 years of experience in the field of sports psychology. Subsequently, another group of translators with more than 8 years of experience in the field of sports psychology translated the items backwards in order to compare the results of their translations with the original items. Once the questionnaire was obtained in Spanish, the items were adapted to a sports context, eliminating terms related to an educational context and replacing them with terms related to a physical exercise or sports context.

Once the final questionnaire was obtained, several secondary schools were contacted and asked to collaborate. The objective of the study was explained to teachers, parents/legal guardians and students. The criteria for participating in the study was the delivery of informed consent from the students’ parents, since the students were minors, and the completion of the questionnaire in full (99.14% of the participants filled out all items). The questionnaire was completed on paper by each individual student before the PE classes, with an approximate duration of 15 minutes. The collection period for all questionnaires was one week, as there were several schools.

The present study followed the postulates established by the protocol of the American Psychology Association, and the approval of the bioethics committee of the University of Almeria was obtained.

### 2.4. Data Analysis

To provide validity tests based on the internal structure, the factor structure of the questionnaire was examined through EFA and CFA. For the CFA, the maximum likelihood method was applied together with the bootstrapping procedure with 5000 interactions because the Mardia coefficient was very high (124.21) [24]. In addition, reliability and descriptive statistical analyses were performed and the invariance of the questionnaire with respect to gender and age was examined.

The following adjustment rates were considered for CFA [25]: χ2/df, with a score of 3 or less acceptable; CFI, NFI, TLI and IFI, with a score of 0.95 or more acceptable; RMSEA, with its CI at 90%, with values below 0.06; SRMR, with values below 0.08. Correlations between factors show an adequate level of conceptual divergence when the upper limit of their 95% CI does not exceed 0.85 as an absolute value [26].

## 3. Results

### 3.1. Exploratory Factorial Analysis

First, with the sample of 847 students, an EFA was carried out using the 15 items that made up the scale, reflecting on the distribution of the items, as shown in Table 1. The results showed three components with eigenvalues higher than 1, which represented 70.39% of the variance.

### 3.2. Descriptive Statistics, Reliability Analysis and Bivariate Correlations

The mean (M), standard deviation (SD) and bivariate correlations are shown in Table 2. The correlations of the three factors showed significant high valence.

As for the reliability analysis, it was calculated through Cronbach’s alpha index, having a score of 0.86 for organizational dimension, 0.84 for procedural dimension and 0.82 for cognitive dimension.

### 3.3. Confirmatory Factor Analysis

**Figure 1:**
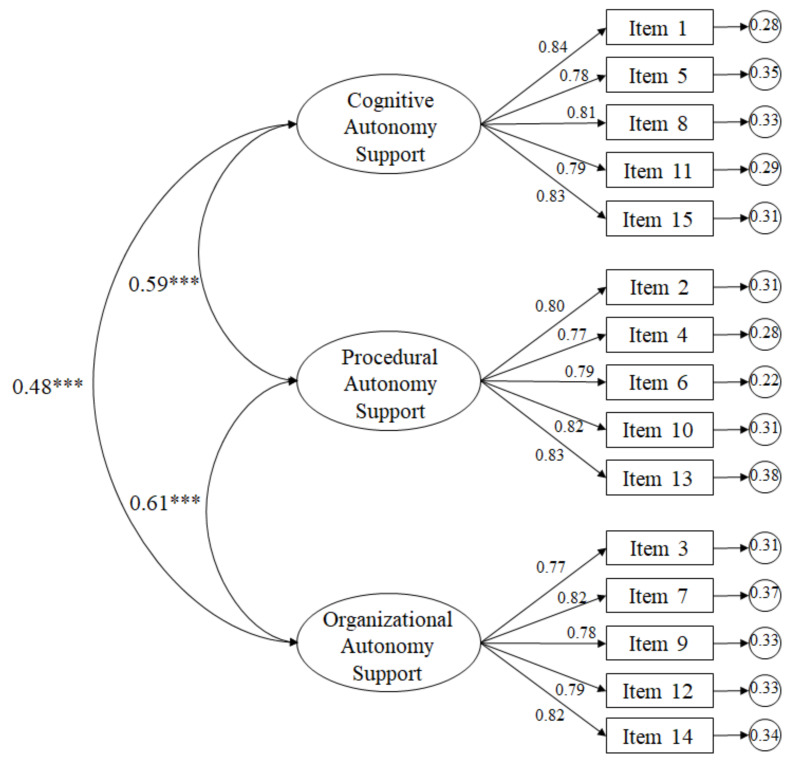
Confirmatory factor analysis of the tested model. *** *p* < 0.001.

The model tested in Figure 1 showed acceptable fit rates: χ2 (87. N = 1482) = 243.11, *p* < 0.001; χ2/df = 2.79; TLI = 0.97; NFI = 0.97; CFI = 0.97; IFI = 0.97; RMSEA = 0.051 (CI 90% = 0.047–0.066); SRMR = 0.044. Standardized regression weights ranged from 0.77 to 0.84 and were statistically significant (*p* < 0.001).

The higher-order model revealed appropriate adjustment rates: χ2 (87. N = 1482) = 231.79, *p* < 0.001; χ2/df = 2.66; NFI = 0.98; IFC = 0.98; TLI = 0.98; IFI = 0.98; RMSEA = 0.046 (CI 90% = 0.039–0.062); SRMR = 0.040. The correlation between the higher order factor (support for autonomy) and the organizational dimension was 0.56, the procedural dimension 0.65 and the cognitive dimension 0.51.

### 3.4. Age and Sex Invariance Analysis

Table 3 and Table 4 show the results of the invariance analysis with respect to age and sex in order to demonstrate whether the factor structure is shown to be invariant in both the three-factor model and the higher-order model. In this sense, the absence of significant differences in the statistic χ2 between model 1 and model 2 is a minimum criterion to satisfy that the questionnaire is understood in a similar way regardless of age and sex [27].

Table 3 and Table 4 show significant differences in gender and sex between models 2 and 3 of the three-factor model. As a higher-order model, significant differences can be observed between models 4 and 5 in both of the analyses of invariance with respect to sex and age. These results show that both men and women had a similar understanding of the questionnaire, regardless of their age.

## 4. Discussion

This study aimed to adapt and validate the Multi-Dimensional Perceived Autonomy Support Scale for Physical Education by Tilga et al. [7] to a Spanish physical exercise or sport context, from the perspective of Self-Determination Theory [28]. For this purpose, the psychometric properties of the Multi-Dimensional Perceived Autonomy Support Scale for Physical Activity (MD-PASS-PA) questionnaire were analyzed. Furthermore, the theoretical postulates of Stefanou et al. [6] were taken into account. These authors considered that the students’ perception of support teacher autonomy during PE classes was divided into three different dimensions: support for organizational autonomy, support for cognitive autonomy and support for procedural autonomy. This structure was later confirmed by Burgueño et al. [22], but as the original scale towards the context of PE. The results showed that the new instrument, MD-PASS-PA, has adequate psychometric properties, being a reliable and valid instrument to measure autonomy support in a multi-dimensional way.

The results of the present study have shown, through the EFA, that the scale is composed of three factors. This result has been subsequently endorsed through CFA. In addition, a second CFA was performed in order to determine the psychometric properties of the higher-order model, showing acceptable adjustment rates. This is interesting because it supports the use of an overall value composed of the mean of the three factors, which can be used by researchers in order to simplify models where several constructs are present. These results are similar to those achieved by Tilga et al. [7] in their original scale through EFA and CFA. Regarding the analysis of invariance through gender and age, the present study showed that the factor structure of the scale was invariant with respect to gender and age, due to the existence of significant differences between model 2 and model 3 in the three-factor model and between models 4 and 5 in the higher-order model. These results are similar to those achieved by Tilga et al. [7] in the original scale, where they showed that their questionnaire was understood in a similar way regardless of the participants’ sex. In this way, the questionnaire will allow future studies to establish comparisons between men and women and between populations of different ages in order to determine differences between populations. Reliability analyses showed a score of above 0.80 for Cronbach’s alpha, which was higher than the original scale [7]. 

Finally, the bivariate correlations between the factors reflected the same valence as in the CFA. In addition, the correlation established in the CFA between the factors was less than 0.85. These results are similar to those of the original scale. Therefore, it is possible to establish that the scale has adequate discriminant validity [19]. Following the results obtained in this study, secondary schools have an effective tool for assessing support for the autonomy of their students in relation to physical activities or sports. 

This instrument will provide the opportunity to obtain more precise and holistic information (procedural, cognitive and organizational dimensions) regarding teachers’ roles and performances towards the creation of active, physical and sports exercise habits in their students [29]. In this sense, several studies from the SDT, which have used the PASSES one-dimensional scale, have shown that support for autonomy has a positive influence on mediating variables, such as basic psychological needs and motivation [12] and on the cognitive (e.g., attention and memory [29]), behavioral (e.g., physical activity practice and diet [30]) and affective (e.g., anxiety and stress [31]) consequences shown by students during PE classes during their teaching and learning process. Finally, this instrument will allow teachers to organize and employ strategies more appropriate to the characteristics and interests of their students. This will not only improve the quality of the methodologies applied during PE classes, but will also increase PE teacher training programs [22].

However, there are a number of limitations that should be highlighted. Firstly, the adaptation and validation of the scale was carried out using a student population from the south of Spain following an incidental non-probabilistic sampling. Secondly, with regard to age groups, only a very restricted range was taken into account, limited to secondary students. In this sense, it would be interesting for future studies to extend this age range, since there are various stages of education (primary and secondary education and university).

## 5. Conclusions

Based on the results achieved in this study, the Multi-Dimensional Perceived Autonomy Support Scale for Physical Exercise, as shown in Appendix A, can be considered a valid and reliable instrument. In this sense, the EFA and the CFA showed adequate psychometric properties and validity. Thus, this instrument can significantly contribute to the clarification of the behavioral processes of teachers in their interactions with students.

## Figures and Tables

**Table 1:** Loadings from the EFA.

Items	Cognitive Dimension	Procedural Dimension	Organizational Dimension
Item 1	0.81		
Item 2		0.82	
Item 3			0.85
Item 4		0.79	
Item 5	0.87		
Item 6		0.78	
Item 7			0.89
Item 8	0.85		
Item 9			0.83
Item 10		0.80	
Item 11	0.86		
Item 12			0.79
Item 13		0.83	
Item 14			0.78
Item 15	0.80		

**Table 2:** Statistics and bivariate correlations.

Factors	*M*	*SD*	Range	1	2	3
1. Organizational Autonomy Support	4.57	1.01	1–7		0.59 **	0.48 ***
2. Procedural Autonomy Support	5.29	0.80	1–7			0.62 ***
3. Cognitive Autonomy Support	4.87	1.15	1–7			

Note: ** *p* < 0.01, *** *p* < 0.001.

**Table 3:** Analysis of invariance by sex.

Three–Factor Model										
Models	χ^2^	*df*	χ^2^/*df*	Δχ^2^	Δ*df*	CFI	TLI	IFI	RMSEA (CI 90%)	SRMR
Model 1	498.99	174	2.87	–	–	0.97	0.97	0.97	0.051 (0.049–0.058)	0.042
Model 2	523.47	186	2.81	11.56	12	0.97	0.97	0.97	0.054 (0.050–0.059)	0.043
Model 3	526.96	192	2.74	43.57 **	18	0.96	0.96	0.96	0.055 (0.051–0.060)	0.047
Model 4	555.61	207	2.68	66.54 ***	33	0.96	0.96	0.96	0.056 (0.054–0.062)	0.049
**High–Order Model**										
**Models**	**χ^2^**	***df***	**χ^2^/*df***	Δχ^2^	**Δ*df***	**CFI**	**TLI**	**IFI**	**RMSEA (CI 90%)**	**SRMR**
Model 1	506.94	174	2.91	–	–	0.97	0.97	0.97	0.055 (0.051–0.062)	0.049
Model 2	532.80	186	2.86	11.56	12	0.97	0.97	0.97	0.055 (0.051–0.063)	0.049
Model 3	529.10	188	2.81	15.88	14	0.96	0.96	0.96	0.056 (0.052–0.063)	0.047
Model 4	516.79	189	2.73	21.07	15	0.96	0.96	0.96	0.056 (0.053–0.064)	0.047
Model 5	511.70	192	2.66	43.57 **	18	0.95	0.95	0.95	0.059 (0.053–0.064)	0.045
Model 6	540.99	207	2.61	66.54 ***	33	0.95	0.95	0.95	0.059 (0.056–0.063)	0.045

** *p* < 0.01; *** *p* < 0.001. Note: Model 1 = unconstrained; Model 2 = measurement weights; Model 3 = structural weights; Model 4 = structural covariances; Model 5 = structural residuals; Model 6 = measurement residuals.

**Table 4:** Analysis of invariance by age.

Three–Factor Model										
Models	χ^2^	*df*	χ^2^/*df*	Δχ^2^	Δ*df*	CFI	TLI	IFI	RMSEA (CI 90%)	SRMR
Model 1	406.25	174	2.33	–	–	0.97	0.97	0.97	0.052 (0.049–0.059)	0.045
Model 2	456.35	186	2.45	26.46	12	0.96	0.96	0.96	0.053 (0.050–0.059)	0.046
Model 3	484.68	192	2.52	37.85 **	18	0.96	0.96	0.96	0.053 (0.051–0.060)	0.046
Model 4	552.68	207	2.67	67.81 ***	33	0.95	0.95	0.95	0.056 (0.053–0.061)	0.048
**High–Order Model**										
**Models**	**χ^2^**	***df***	**χ^2^/*df***	**Δχ^2^**	**Δdf**	**CFI**	**TLI**	**IFI**	**RMSEA (CI 90%)**	**SRMR**
Model 1	519.91	174	2.99	–	–	0.97	0.97	0.97	0.057 (0.053–0.063)	0.048
Model 2	549.33	186	2.95	12.80	12	0.97	0.97	0.97	0.057 (0.053–0.063)	0.048
Model 3	542.25	188	2.88	13.82	14	0.96	0.96	0.96	0.057 (0.053–0.063)	0.046
Model 4	528.97	189	2.80	13.91	15	0.95	0.95	0.95	0.059 (0.055–0.064)	0.046
Model 5	539.03	192	2.81	41.39 **	18	0.95	0.95	0.95	0.059 (0.055–0.064)	0.044
Model 6	559.18	207	2.70	55.77 **	33	0.95	0.95	0.95	0.060 (0.056–0.065)	0.044

** *p* < 0.01; *** *p* < 0.001. Note: Model 1 = unconstrained; Model 2 = measurement weights; Model 3 = structural weights; Model 4 = structural covariances; Model 5 = structural residuals; Model 6 = measurement residuals.

## Appendix A

This scale was validated in Spanish.

**Cognitive Autonomy Support**

1. Mi profesor de educación física se muestra interesado cuando comparto mis experiencias cuando realizo ejercicio físico o deportivo

*My physical education teacher is interested when I share my experiences when I do physical exercise or sports.*

5. Mi profesor de educación física me escucha y responde cuando expreso mi opinión sobre el ejercicio físico o deportivo que realizo en mi tiempo libre.

*My physical education teacher listens and responds to me when I express my opinion about the physical or sports exercise I do in my free time.*

8. Mi profesor de educación física respeta el ejercicio físico o deportivo que realizo en mi tiempo libre.

*My physical education teacher respects the physical exercise or sport I do in my free time*.

11. Mi profesor de educación física me permite expresar mi opinión sobre el ejercicio físico o deportivo que realizo en mi tiempo libre

*My physical education teacher allows me to express my opinion about the physical exercise or sport I do in my free time*.

15. Mi profesor de educación física me transmite seguridad para que pueda realizar ejercicio físico o deportivo en mi tiempo libre

*My physical education teacher gives me security so that I can do physical exercise or sports in my free time*.

**Procedural Autonomy Support**

2. Mi profesor de educación física me guía en la búsqueda del ejercicio físico o deportivo acorde a mis intereses.

*My physical education teacher guides me in my search for physical exercise or sports according to my interests*.

4. Mi profesor de educación física da una visión general sobre los diferentes tipos de ejercicio físico o deportivo que puedo realizar en mi tiempo libre.

*My physical education teacher gives me an overview of the different types of physical or sports exercise I can do in my free time*.

6. Mi profesor de educación física explica por qué tengo que hacer ejercicio físico o deportivo en mi tiempo libre.

*My physical education teacher explains why I need to do physical exercise in my free time*.

10. Mi profesor de educación física me ofrece consejos para seleccionar el ejercicio físico o deportivo que mejor se adapta a mí.

*My physical education teacher gives me advice on selecting the physical exercise or sport that is best for me*.

13. Mi profesor de educación física explica el efecto que tiene el ejercicio físico o deportivo en mí.

*My physical education teacher explains the effect that physical exercise or sport has on me*.

**Organizational Autonomy Support**

3. Mi profesor de educación física me permite escoger diferentes opciones de ejercicios físico o deportivos en mi tiempo libre.

*My physical education teacher allows me to choose different exercise or sports options in my free time*.

7. Mi profesor de educación física confía en mi capacidad para buscar soluciones en relación al ejercicio físico o deportivo que realizo en mi tiempo libre

*My physical education teacher is confident in my ability to find solutions for physical exercise or sports in my free time*.

9. Mi profesor de educación física me permite elegir distintas opciones de ejercicio deportivo.

*My physical education teacher allows me to choose different sports exercise options*.

12. Mi profesor de educación física me permite elegir distintas opciones de ejercicio físico.

*My physical education teacher allows me to choose different options for physical exercise*.

14. Mi profesor de educación física me muestra diferentes tipos de ejercicio físico o deportivo en función del lugar donde se practica.

*My physical education teacher shows me different types of physical exercise or sport depending on where it is practiced*.
